# Fitness testing in tennis: Influence of anthropometric characteristics, physical performance, and functional test on serve velocity in professional players

**DOI:** 10.1371/journal.pone.0259497

**Published:** 2021-11-29

**Authors:** Alejandro Sánchez-Pay, Jesús Ramón-Llin, Rafael Martínez-Gallego, David Sanz-Rivas, Bernardino Javier Sánchez-Alcaraz, Sergio Frutos

**Affiliations:** 1 Human Performance and Sports Science Laboratory, Faculty of Sport Sciences, University of Murcia, Murcia, Spain; 2 Department of Physical Activity and Sport, Faculty of Sport Sciences, University of Murcia, Murcia, Murcia, Spain; 3 Department of Musical, Plastic and Corporal Expression, University of Valencia, Valencia, Spain; 4 National and International Tennis Coach (Level III), Madrid, Spain; Universidad de Extremadura Facultad de Enfermeria y Terapia Ocupacional, SPAIN

## Abstract

The aims of this study were to examine the relationship between anthropometric variables, physical performance, and functional test with serve velocity regarding tennis players’ level and to design regression models that effectively predict serve velocity. A sample of sixteen male tennis players participated in this study (national level = 8, professional level = 7). Anthropometric measurements (body mass, height, body mass index and body segments) and physical test (hand strength, countermovement jump, jump on serve, and serve velocity) and functional test (medicine ball throw overhead and shot put) were performed. No differences in anthropometrics and physical test were found between national and professional levels. A significant positive correlation (*p* < 0.05, ranging for 0.603 to 0.932) was found between some anthropometrics measurements (body mass, height, arm, forearm, and leg segments), physical parameters (hand strength, countermovement jump) and functional test (medicine ball throw shot put and overhead) with serve velocity for all tennis players. Multiple regression analysis indicated that medicine ball throw shot put was the most important test to explain serve velocity (*r*^*2*^ = 0.869). The results showed how the combination of physical and anthropometric factors have an impact on serve velocity. In addition, a new functional fitness test (medicine ball throw shot put) is proposed as an alternative to traditional medicine ball throw overhead due to its high reproducibility (inter-trial reliability) and predictive validity values, as well as by multi-segmental coordination movement similar to tennis serve.

## Introduction

Tennis has evolved in recent years thanks to the evolution of materials for the manufacture of rackets and balls, and better quality training [1]. It is presently characterized by speed, power, and strength, with higher stroke and serve velocities, which makes the service a key factor in game success [2]. Tennis serve has been described as the most potentially dominant stroke in the modern game [3, 4]. An increasing serve speed reduces the time for the opponent to return the ball successfully and increases the probability of the server’s superiority in the following game or of gaining a direct point [4–6].

**Fig 1:**
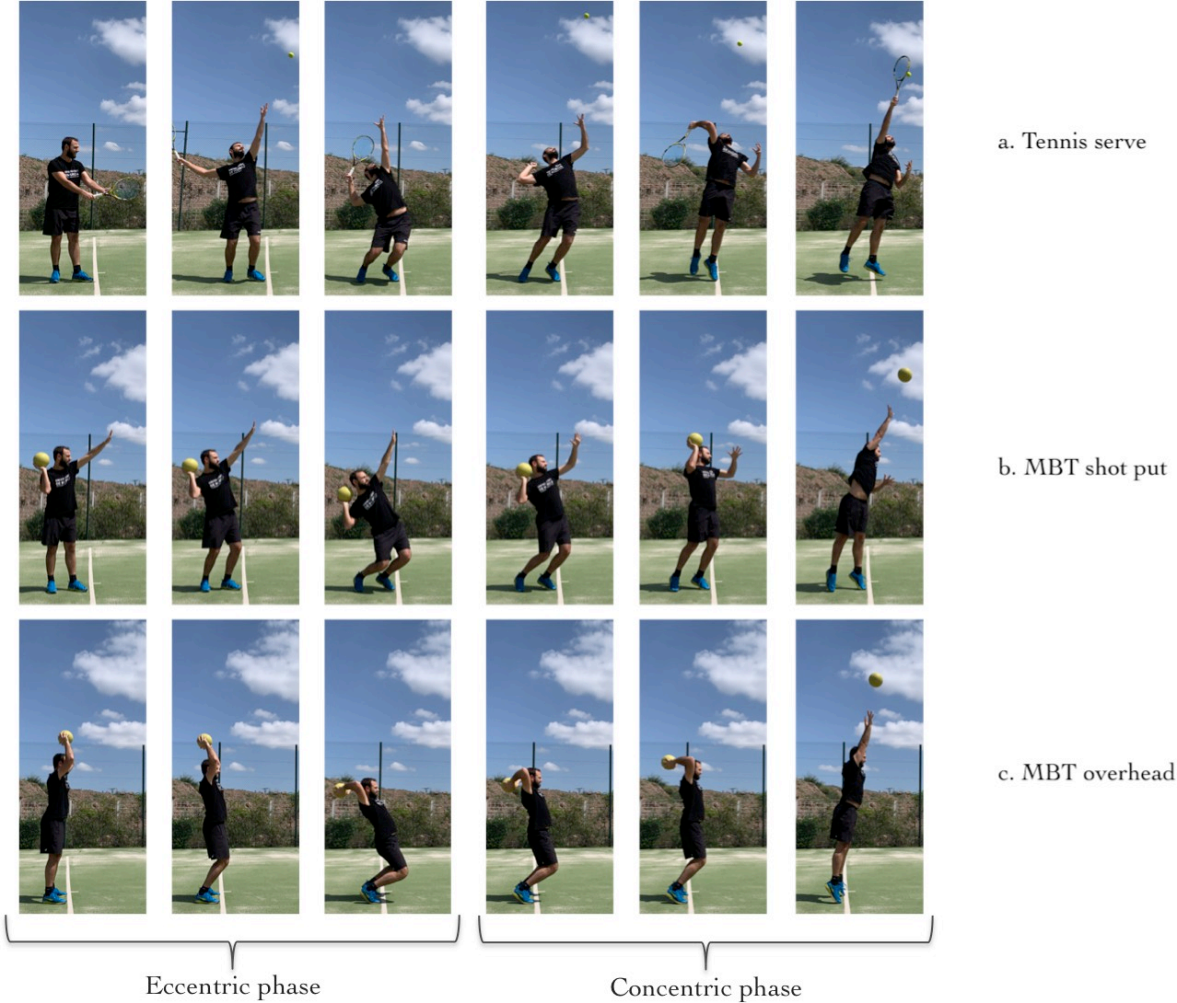
Sequence of movements of tennis serve and Medicine Ball Throw (MBT) overhead and shot put.

Given the importance of the speed of the serve, finding exercises that give a greater transfer to the speed of this or anthropometric parameters of the players who achieve high service speeds, will help to improve sports training. Many studies have observed the relationship of serve velocity (SV) with some parameters such as anthropometric [5, 7], technique [8] or physical conditioning [1]. Research has been conducted with junior [1, 9–12] and professional tennis players [13], using different isometric and dynamic strength tests with the aim of identifying the most influential factors on SV [14, 15]. Regarding upper-body power tests, the medicine ball throw (MBT) overhead has been used as a possible predictor for SV [1, 10, 11]. Results are diverse, because the coordination of body segments (ie, kinetic chain) during MBT and tennis serve strokes seem different (Fig 1a–1c). However, because of the serve’s great influence on match outcome, identifying specific predictors of SV could be a crucial aspect to design effective training programs [1].

From a biomechanical point of view, serve is the most complex movement in tennis. Producing a high speed tennis serve requires a proper kinetic chain which involves sequential activation and coordination of different body parts (leg, trunk, shoulder, elbow, and wrist) [16, 17]. Kinetic energy in tennis serve is produced almost equally between the upper extremity and lower extremity throughout the motion [8]. Serve biomechanical requirements can be specifically analysed using an 8-stage model (star, release, loading, cocking, acceleration, contact, deceleration, and finish) [3]. Loading stage of the lower body has been described as the ‘loaded position’ where the dominant elbow is the lowest vertical position, and the maximal knee flexion [3] exists and occurs at the end of the eccentric phase of the movement (Fig 1a). Knee flexion before extension is a prerequisite for an efficient execution of the serve [18]. Getting to generate vertical force in that phase, will allow the player to have a greater impact height [19]. A higher impact height is a positive correlation to the SV, either by higher body height [5, 12, 13] or by jumping higher [20].

It is reasonable to assume that physical conditioning (muscle power and strength) and anthropometric data (height, body mass index and body segment length) may affect serve performance [1]. However, there is a lack of consensus regarding scientifically proven predictors of SV because serve movement appears to be different depending on the players’ level or age [21, 22]. We hypothesised that some of physical and anthropometric parameters would be a relation to SV. In addition, due to the service is a multi-segmental coordination mechanism and the MBT shot put appears to have a similar pattern of movement could show a high correlation with hitting speed for tennis players who have a stable pattern (high level). Thus, the aims of this study were (1) to examine the relationship between physical performance, anthropometric and functional test with SV regarding tennis players’ level and (2) to design regression models that effectively predict SV based on the relationship between these variables. In this way, the results of this study will provide relevant information for specific training based on serve stroke in high-level male tennis players.

## Methods

### Participants

Non-randomized sampling was carried out in an international tennis tournament. Fifteen adult male tennis players (mean ± SD; age: 19.66 ± 1.63 years) agreed to take part in this study. Players were classified according to their level of competition in two groups: professional level (PL) (n = 7; ranking = 300–900 ATP Ranking) and national level (NL) (n = 8; ranking = 400–900 Spanish National Ranking). Written informed consent was obtained from all participants. The study was approved by the Royal Spanish Tennis Federation Committee and all procedures conform to the recommendations of the Declaration of Helsinki of 2013. The individual pictured in Fig 1 has provided written informed consent (as outlined in PLOS consent form) to publish their image alongside the manuscript.

### Procedures

**Fig 2:**
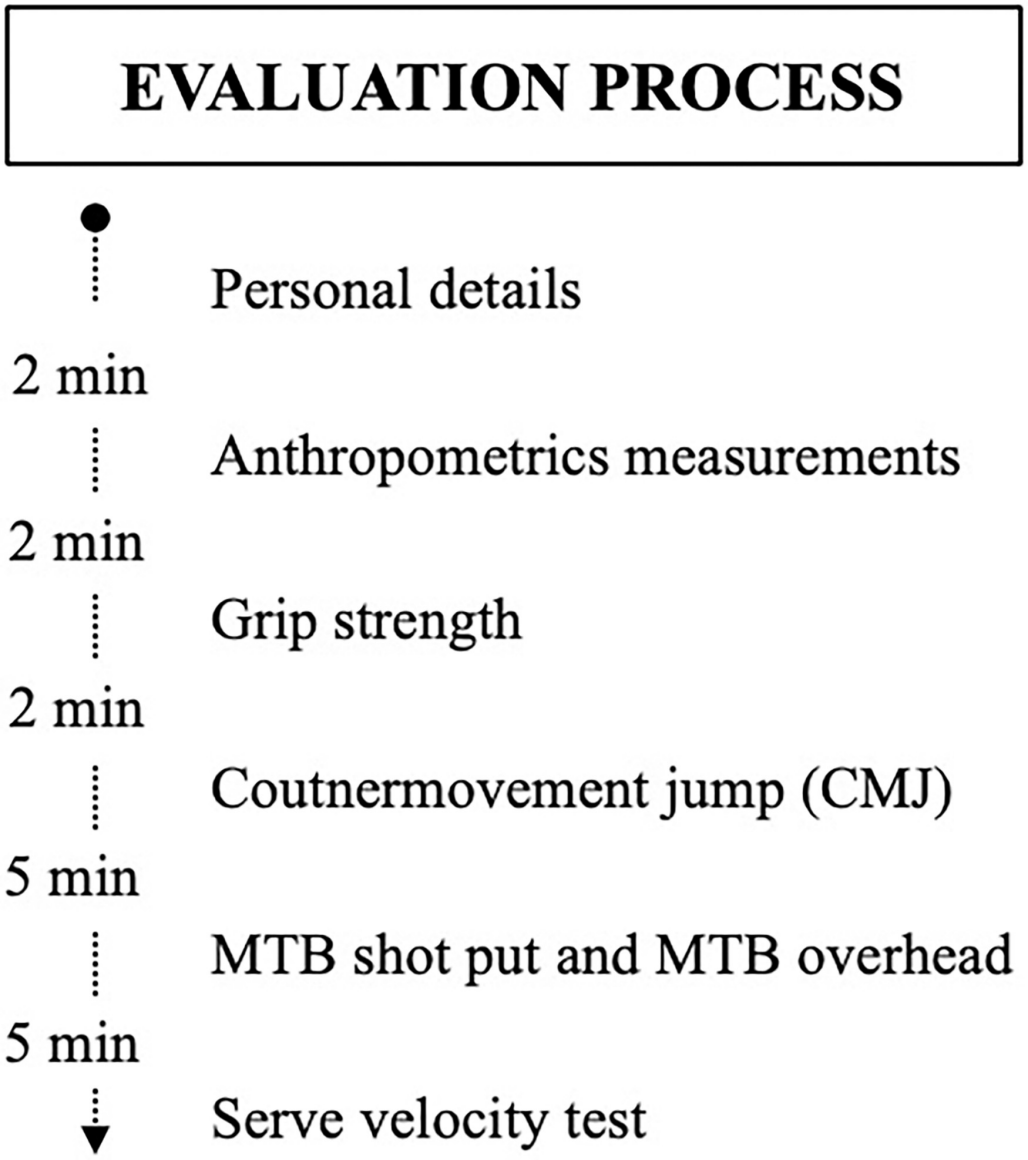
General evaluation process.

Testing protocols were conducted in the same day, during an International Tennis Tournament (ITF Future 15.000$). To ensure standardization of test administration all tests were performed into the same order, using the same testing devices, measurement protocols and operators (Fig 2). The test session was performed in an outdoor synthetic tennis court (Green Set^®^ surface; temperature, 22.1–26.8°C; relative humidity, 54.4–67.2%; Kestrel 4000 Pocket Weather Tracker, Nielsen Kellerman, Boothwyn, PA, USA), between 9:00 and 14:00 hours, 24 h after the last training session and 2 h post-prandial. A specific dynamic warm-up routine was carried out before the tests, consisting of skipping rope, dynamic stretching, hopping exercises, jumps of increasing intensity and tennis serves. Each test session lasted approximately 30–40 minutes. All participants were familiarized with the tests before the evaluation began and they could hydrate at will in breaks between tests. No injuries or incapacities were reported.

#### Anthropometry

Following familiarization, anthropometry data were collected on all participants including height using a fixed stadiometer (± 0.1 cm; Seca 220, Hamburg, Germany), weight using a digital balance (± 0.1 kg; OK OPS 100, Ingolstadt, Germany), and length of body segments (arm, forearm, thigh and calf) using an anthropometer [23]. Body mass index was calculated using the formula BMI = weight [kg] / height [m^2^]. All these measures were obtained by trained and experienced evaluators, following the standardised techniques adopted by the International Society for the Advancement of Kineanthropometry (ISAK) and Norton et al. (1996) [24].

#### Grip strength

Maximal handgrip strength was measured with a portable hand dynamometer Smedley III T-18A (Takei, Tokyo, Japan). Hand dynamometer has a range between 0 and 100 kilogram (kg) with 0.5 kg increments and an accuracy of ± 2 kg. Dominant handgrip strength test was performed in a standing position, with the elbow extended, and the arm positioned with the dynamometer parallel to the subject’s side. Participants were asked to perform a maximal voluntary contraction, squeezing the dynamometer as hard as possible, for 3 s [25]. The maximum force (kilogram) achieved 2 trials for each side was recorded. Resting time between attempt was 2 min.

#### Countermovement jump (CMJ)

To assess lower-limb explosive power, a double leg vertical CMJs without arm swing (i.e., with the hands on the hips) was performed on a contact time Optojump platform (Microgate^®^, Bolzano, Italy) according to established protocol [26]. Each player performed 2 maximal attempts interspersed with 45 s of passive recovery, and the maximum height (cm) determined by flight time was recorded [25].

#### Medicine ball throw overhead and shot put

Holding a 2-kg medicine ball (Fitness-MAD 2Kg, Evesham, United Kingdom), the players stood at a line facing the throwing direction with the feet side-by-side and slightly apart. After the ball was brought back behind their head with two hands, it was thrown forward as far as possible without moving the feet and crossing the line, to perform overhead MBT (Fig 1c). To perform shot put MTB, the ball was held on the palm of the dominant hand. Then the ball was brought to the side of the head, bending knees while keeping the no dominant arm raised up. After that, the medicine ball was thrown forward as far as possible without crossing the line and moving the feet (Fig 1b). The distance was measured between the starting line and the point where the ball landed. Each player performed 2 repetitions, and the best performance was recorded to the nearest 5 cm [1, 25].

#### Service velocity test

First serve velocity was measured using a standard radar gun (Stalker Pro Inc., Plano, Texas, USA). Radar gun radar has a data recording frequency of 33 hz and measures with a speed range from 1 to 480 km/h (± 0.16 km/h). The radar was situated 4 m behind the server, at the center of the baseline, and was aligned to the height of 3 m, pointing down the center of the court [1]. Players were able to use their own rackets in order to achieve a more accurate result. Right-handed players served from the deuce court and left-handed players served from the ad court. Players performed 2 sets of 8 first serves, building up to their maximum speed, with 10 seconds rest between serves and 2 min rest between series. Serve direction was down the “T” (center line), with new balls used for each serve [1].

To be accepted, serves had to fall into the service box. The researcher provided direct velocity feedback to encourage maximal effort [9]. The peak velocity of each stroke was recorded. For further analysis, the average velocity of all serves was used (Fett et al., 2020; Ulbricht et al., 2013). Players served on a contact time Optojump platform (Microgate^®^, Bolzano, Italy) and flight time was also measured in each serve [20].

### Statistical analyses

Exploratory data analysis included mean and standard deviation descriptive statistics, searching for outliers and assessing the normality of distribution by means of Shapiro–Wilk tests. Levene’s test was used to test the equality of variances. The reliability of test measurements (Table 1) were assessed using Intraclass Correlation Coefficients (ICCs), the standard error of measurements (SEM), and the coefficient of variation (CV). Student’s t-test was used to determine the possible differences of each variable according to players’ level (national-professional). Effect sizes (d) were estimated by calculating Hedges’ g due to sample size [27]. Effect sizes were interpreted for trained players (+ 10 years of experience) as follows [28]: Trivial (0–0.25), Small (0.25–0.50), Moderate (0.50–1.0), and Large (> 1.0). Pearson correlations analysis was used to detect potential confounders between fitness performance variables and SV among tennis players. The average service velocity was used as the main variable due to: a) this variable showed a higher correlation than the maximum service velocity variable; and, b) average service velocity has been used as the main variable in other studies [1, 25]. Correlations were classified as Trivial (0–0.1), Small (0.1–0.3), Moderate (0.3–0.5), Large (0.5–0.7), Very large (0.7–0.9), Nearly perfect (0.9), and Perfect (1.0) [29]. Multiple linear regressions (stepwise) were used to identify predicting factors for the SV [1, 30]. Average SV was used as the dependent variable in the multiple regression analysis, whereas the variables of anthropometric and fitness performance measurements operated as independent predictors. The significance level was set to *p* < 0.05. Data analyses were performed using SPSS statistical software package (version 20.0; SPSS, Inc., Chicago, IL, USA).

**Table 1:** Inter-trial reliability of test measurements.

	ICC (95% CI)	SEM	CV (%)
Grip strength (kg)	0.933 (0.808–0.977)	2.96	7.21
CMJ (cm)	0.974 (0.924–0.991)	0.01	2.02
MBT Shot put (m)	0.949 (0.852–0.983)	0.71	6.07
MBT Overhead (m)	0.970 (0.912–0.990)	0.56	6.48
Avg. Service velocity (km·h-1)	0.989 (0.979–0.996)	6.51	3.88
Jump on service (s)	0.648 (0.183–0.906)	0.10	38.41

ICC: Intraclass correlation coefficient. CI: Confidence interval. SEM: standard error of measurement (calculated as the square root of the root mean). CV: Coefficient of variation. CMJ: countermovement jump. MBT: Medicine ball through. Avg.: Average.

## Results

Anthropometric and fitness parameters are shown in Table 2. Professional level players showed higher values in all parameters (except in MBT shot put), although no statistically significant differences were found between level groups (*p* > .05). Arm and thigh length were the anthropometric parameters with higher differences between levels. Grip, CMJ, MBT Overhead and jump on service were the parameters that showed to be closer to significant differences.

**Table 2:** Anthropometrics and fitness parameters in tennis players.

Anthropometrics	National	Professional	p value	Dif	g Hegges	ES
	n = 8	n = 7				
	mean (s.d)	mean (s.d)				
Body mass (kg)	72.58 (11.41)	77.79 (7.31)	0.320	5.21	0.50	Moderate
Height (m)	1.76 (0.12)	1.81 (0.05)	0.288	0.05	0.50	Moderate
BMI (kg·m-2)	23.38 (2.1)	23.68 (1.53)	0.758	0.30	0.15	Trivial
Arm (cm)	35.48 (2.19)	37.13 (1.29)	0.105	1.65	0.85	Moderate
Forearm (cm)	27.79 (1.81)	28.4 (1.43)	0.484	0.61	0.35	Small
Thigh (cm)	40.69 (2.06)	42.53 (3.36)	0.217	1.84	0.63	Moderate
Leg (cm)	40.14 (6.3)	42.69 (1.76)	0.321	2.55	0.50	Small
**Fitness**						
Grip strength (kg)	42.19 (7.19)	48.36 (8.99)	0.163	6.17	0.72	Moderate
CMJ (cm)	0.31 (0.06)	0.35 (0.05)	0.148	0.04	0.68	Moderate
MBT Shot put (m)	12.19 (3.03)	12 (1.14)	0.883	-0.18	-0.08	Trivial
MBT Overhead (m)	9.11 (2.35)	11.24 (2.14)	0.092	2.12	0.89	Moderate
Avg. Service velocity (km·h-1)	169.63 (20.95)	172.84 (12.91)	0.731	3.21	0.17	Trivial
Max. Service velocity (km·h-1)	174.5 (20.78)	180.29 (14.37)	0.548	5.79	0.30	Small
Jump on service (s)	0.25 (0.05)	0.28 (0.05)	0.238	0.03	0.56	Moderate

BMI = body mass index; CMJ = countermovement jump; MBT = medicine ball throw; Avg = average; Max = Maximal; s.d. = standard deviation; Dif. = difference between means; ES = Effect Size

The correlation coefficients of the anthropometric and fitness parameters with SV average by level are presented in Table 3. For all of the players, the highest correlation was observed in MBT shot put 0.932 (nearly perfect). Body mass, height and forearm length were the anthropometrics parameters with a very long effect size (from 0.776 to 0.851). For national level players, almost all anthropometrics and fitness parameters showed a very large and nearly perfect correlation with service velocity (from 0.746 to 0.983). In professional level players, MBT shot put was the variable with the highest correlation (r = 0.825).

**Table 3:** Correlation coefficients of anthropometric and fitness characteristics with medium serve velocity.

Anthropometrics	National		Professional		Total players	
	n = 8		n = 7		n = 15	
	r	p	r	p	r	p
Body mass (kg)	0.934	0.001	0.337	0.459	0.776	0.001
Height (cm)	0.914	0.002	0.692	0.085	0.851	<0.001
BMI (kg·m-2)	0.330	0.425	-0.086	0.854	0.220	0.431
Arm (cm)	0.818	0.013	0.007	0.988	0.603	0.017
Forearm (cm)	0.858	0.006	0.607	0.148	0.780	0.001
Thigh (cm)	-0.746	0.034	0.554	0.197	-0.087	0.759
Leg (cm)	0.773	0.025	0.236	0.611	0.677	0.006
**Physical test**						
Grip strength (kg)	0.896	0.003	0.326	0.475	0.618	0.014
CMJ (cm)	0.847	0.008	0.362	0.425	0.664	0.007
MBT Shot put (m)	0.983	<0.001	0.825	0.022	0.932	<0.001
MBT Overhead (m)	0.746	0.034	0.501	0.252	0.626	0.013
Jump on service (s)	0.205	0.626	0.643	0.168	0.338	0.238

BMI = body mass index; CMJ = countermovement jump; MBT = medicine ball throw;

r = Pearson correlation

Results of the multiple regression analysis for anthropometric and fitness parameters depending on the level group are shown in Table 4. In the main model for all players, MBT shot put explained 87% of service velocity (*r* = 0.932, *r*^*2*^ = 0.869, *p* < 0.001). In addition, a second model with MBT shot put and forearm parameter explained 93% of service velocity for all players (*r* = 0.962, *r*^*2*^ = 0.925, *p* < 0.001). The model for national level players was explained almost perfectly (97%) through the parameter MBT shot put (*r* = 0.983, *r*^*2*^ = 0.966, *p* < 0.001), and a second model with MBT shot put and height explained 99% of service velocity (*r* = 0.997, *r*^*2*^ = 0.994, *p* < 0.001). For professional level players, MBT shot put was the most important variable, which explains 68% of the predictor model (*r* = 0.825, *r*^*2*^ = 0.680, *p* < 0.001).

**Table 4:** Statistics of multiple regression analysis.

**All Players**	R	R^2^	R^2^ adjust	F	Sig F.
*Model 1*	0.932	0.869	0.858	85.640	< 0.001
			Beta	T	Sig T.
MBT shot put			0.932	9.254	< 0.001
**All Players**	R	R^2^	R^2^ adjust	F	Sig F.
*Model 2*	0.962	0.925	0.913	74.861	< 0.001
			Beta	T	Sig T.
MBT shot put			0.733	7.170	< 0.001
Forearm			0.312	3.052	0.010
**National level**	R	R^2^	R^2^ adjust	F	Sig F.
*Model 1*	0.983	0.966	.962	177.052	< 0.001
			Beta	T	Sig T.
MBT shot put			0.983	13.306	< 0.001
**National level**	R	R^2^	R^2^ adjust	F	Sig F.
*Model 2*	0.997	0.994	.990	356.955	< 0.001
			Beta	T	Sig T.
MBT shot put			0.734	10.661	< 0.001
Height			0.297	4.309	0.008
**Professional level**	R	R^2^	R^2^ adjust	F	Sig F.
*Model 1*	0.825	0.680	0.616	10.616	0.022
			Beta	T	Sig T.
MBT shot put			0.825	3.258	0.22

MBT = medicine ball throw; R^2^ adjust = R^2^ adjusted to the number of the model´s predictors; Sig F = Significance value of the ANOVA test; Sig T = Significance value of the T test

## Discussion

Tennis serve has been considered the most important stroke in professional modern tennis, but the multifactorial nature of the action, makes it very difficult to establish the factors that affect it. Therefore, this study aimed to analyse whether anthropometric variables, physical performance and functional test are related to SV. Furthermore, design regression models predicted SV by level groups in professional tennis players including a new MBT test.

In line with our hypothesis, a nearly perfect correlation was observed in MBT shot put with SV (Table 3) for the analysis of all players regardless of the level of play. Besides in the multiple linear regression analysis, the MBT shot put was the main variable of the predictive model, which explained 87% of SV (Table 4). This means that 87% of the variance in the SV can be predicted by the MBT shot put records. Moreover, this test showed high inter-trial reliability (ICC = 0.949) and predictive validity values (Tables 3 and 4). No study using this test has been found in professional tennis players; therefore, the results cannot be compared with other studies. A similar test (to throw a 200 gr ball) has been used to measure physical performance in juniors, comparing them by sex and age [31, 32] and it was not valid to predict future tennis performance of junior elite tennis players [11]. Also, the diameter of the ball (6.5 cm) similar to an official tennis ball, would allow a throwing motion that involves several significant mechanical differences with serve movement [33] and there would be no need “to push” from the bottom up.

The MBTs require the ability to transfer power from the lower to upper limbs [1]. The MBT (simulating forehand and backhand strokes) has been used to measure strength in tennis players [34] showing a high correlation with trunk rotation and flexion strength [35] and the speed of forehand shot and backhand accuracy shot [36]. Conversely, MBT overhead (frequently used to predict the speed of the serve) which includes a large activation of rectus abdominis (used in tennis serve), has not found a very high correlation in junior (*r* = 0.12–0.60) [1, 9, 10] or adult tennis players [37]. In our study, the MBT overhead significantly correlated with the SV (Table 3), although it was not considered in the predictive model. The differences between the poor correlation in junior and a high correlation in our study could be determined by the age of the participants. Performance on a MBT requires motor coordination performance and kinematic patterns which are different depending on age and performance of tennis players [21, 38, 39].

There was a significant correlation between body height and peak serve speed (*r* = 0.851), which follows the line of previous research in male junior [9, 12] and professional male and female tennis players [5, 13]. It could be hypothesized that taller players have longer body segments, so they could have a more powerful kinetic chain. Moreover, taller players have an advantage in being able to hit the ball at a higher height with a larger service area into which the ball can land [5]. Also, a player can hit the ball higher through a jump before impact. To do this, it is necessary to have great power in the lower limbs. The results in this study showed a moderate correlation between the CMJ and the SV (*r* = 0.664). The results found in other studies show discrepancies on whether there is [12] or not [1, 10, 13] a relationship between CMJ test and SV. A test where the height of the jump is measured during the serve could be more practical; Dossena et al. (2018) measured this (through flight time), showing a slightly positive correlation with SV (*r* = 0.71). In this study no positive correlation was found (Table 3). This could be due to the fact that take-off and landing of the feet during the serve is different among players, so it would not be correct to relate it to the height reached, but to the flight time [40]. This diversity of results questions whether the vertical jump performance reflects lower limb activity during tennis stroke production [41] so more research is needed in this area.

Regarding handgrip strength, the results of this study found an association between handgrip and serve speed among national and all players groups (*r* = 0.896 and *r* = 0.618) although not among international level players (*p* > 0.05). Handgrip strength has positively correlated with SV in junior players [14] although more strongly in male than in females [1]. The wrist represents the final link of this kinetic chain, not creating the power, but transferring the final ball speed [14]. This could explain the lack of correlation between handgrip and SV in international players, so it could be thought that they have a better transfer of forces than lower ranking players.

Focusing on anthropometric parameters, the body weight variable showed a significantly high relationship with the SV (*r* = 0.776). This relationship is similar to other studies in junior [1, 9, 12] and adults tennis players [5, 12, 42]. This could be explained with the production of torque, since an increase in body mass would increase the torque [42], which in turn would increase the service speed. In relation to the body segment; arm, forearm and leg showed a large to very large correlation with SV (Table 3). The role of the forearm is involved in transmitting the segmental speed of the elbow extension and also, inside it is the muscles responsible for flexing the wrist with a high relationship with the SV [15]. Greater angular momentum of the forearm will increase the forward linear speed of the wrist and therefore plays an important role in accelerating the racket and consequently increasing the speed of the ball [43]. The tennis player’s forearm is understood as a moment arm with the axis of rotation at the elbow, understanding that increasing the length of this moment arm (forearm length) can increase tangential speed [44, 45].

This study was strengthened by the novelty of the sample (professional tennis players), multifaceted evaluation performed (anthropometric, power, strength and SV), and the use of a new designed test (MTB shot put) that can be recommended as a valid and reliable indicator for tennis SV. Furthermore, it is important to note that the use of field tests enhance replicability and applicability to the training practice [46]. This research was limited by the small sample size (n = 15) and the inability to make causal inferences. Furthermore, the sample only included male players, so it would be interesting to include female players as well as observe differences according to age and level. Despite these limitations, the obtained results are valuable and add relevant insights on physical fitness performance and anthropometric variables and their influence on SV in professional tennis players.

Because of the very limited information available about physical and anthropometrics components in professional tennis players, it is essential to perform fitness testing to identify determinant factors in game performance and competition success (i.e. SV). In summary, the results presented demonstrate that some anthropometric parameters (body mass, height and arm, forearm and leg segments length) and physical performance measures (grip strength, CMJ and MBT) correlated positively with SV in professional tennis players. The main finding included a new functional movement through MBT shot put that explained 87% of SV. This result shows the importance of using movement specific testing patterns when attempting to predict ball velocity during the tennis serve.

The use of MBT in sports training is growing as practitioners see the wide range of skills that can be trained or simulated, so strength and conditioning coaches could use medicine balls to train the specific biomechanical variables required for success in their particular sport [47]. The MTB shot put test is presented as a reliable test to evaluate an analogous total-body movement pattern similar to the kinetic chain to tennis serve.

## Conclusion

Producing a high speed tennis serve requires a proper kinetic chain which involves sequential activation and coordination of different body segments (leg, trunk, shoulder, elbow, and wrist) [16]. Results of the multiple regression analysis explained 86% of SV, with MBT shot put showing the importance of using movement specific testing patterns when attempting to predict ball velocity during the service. Usually, the coaches and physical trainers do not have isokinetic devices or force platforms readily available for player assessment. MBT shot put test showed high levels of repeatability (inter-trial reliability) and predict validity. Due to its low price and practical use, we encourage coaches and physical trainers to use the MBT as a practical exercise to improve the kinetic chain of the serve, as well as a physical test (e.g., to analyse the influence of biomechanics aspects in the execution or to assess the athlete’s status), and after specific interventions (e.g., resistance training programs).

## Supporting information

S1 Data (SAV)
